# Supplementary material for: Self-driving lab discovers principles for steering spontaneous emission beyond conventional Fourier optics
Source: Nat Commun. 2025 Dec 9;17:204. doi: 10.1038/s41467-025-66916-0 (PMC12779997; doi:10.1038/s41467-025-66916-0)
Supplement: Supplementary file 1 — Supplementary Information [file 41467_2025_66916_MOESM1_ESM.pdf]

Supplementary Information

Self-driving lab discovers principles for steering spontaneous emission

Saaketh Desai, Sadhvikas Addamane, Jeffery Y. Tsao, Igal Brener, Remi Dingreville, Prasad P. Iyer*
Center for Integrated Nanotechnologies, Sandia National Lab, Albuquerque NM, USA
*ppadma@sandia.gov

Table of Contents

1. Ultra-fast optical dual pump experiment and Semiconductor Metasurface Properties
2. Benchmarking the ML models
    a. Generative capability of the VAE
    b. Efficiency of the active learning
    c. Equation learner network fits and equations at other angles
3. Steering over wide field of view with lens and grating

1. Ultra-fast optical dual pump experiment and Semiconductor Metasurface Properties

We steer incoherent light from a reconfigurable semiconductor (GaAs) metasurface under structured optical pumping, see Figure S1a. We design the metasurface resonance to achieve reconfigurable phase response in reflection under free-carrier excitation (with optical pumping). Additionally, the metasurface resonances are aligned to the embedded InAs quantum dot emitters ($\lambda_e = 1280nm$)) such that the photoluminescence (PL) peak and the reflection peak are spectrally overlapping. The metasurfaces where designed such that under the influence of the optical pump induced refractive index change, the phase (ϕ) of the light in reflection undergoes a $0 - 2\pi$ phase shift with minimal change in the amplitude. We demonstrate that this design criteria constructed for coherent reflection translates into momentum change for the light emission under spatially structured optical pumping. The GaAs metasurfaces were grown with a reflective distributed Bragg grating made up of 15 pairs of $Al_{0.3}Ga_{0.7}As$ and AlAs layers with $\lambda_e/4n$ thicknesses, where n is the refractive index of each of the layer (3.2 and 2.94 respectively) at the emission wavelength[1,2]. InAs quantum dots (QDs) were also epitaxially grown within the top GaAs layer as dot in a well (DWELL) configuration.[3] The metasurfaces where fabricated using traditional nano-fabrication techniques which included:

a) Electron beam lithography to define the metasurface resonator shape (width = 280nm) and periodicity (400mn).
b) Deposition and lifted-off an Al_2O_3 hard mask of 25nm.
c) Dry etching 675nm of the top GaAs layer using a Cl_2 gas etch chemistry.

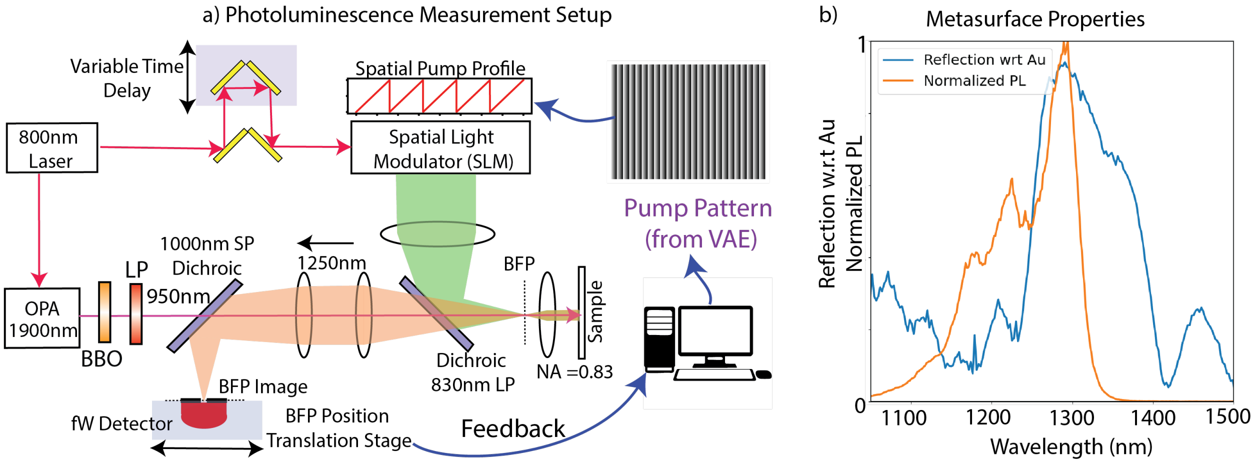

Figure S1a) The photoluminescence steering setup b) The reflection (blue) and photoluminescence (orange) spectra measured for the fabricated metasurface.

d) Verifying the fabrication process using scanning electron microscope images, see Figure 1a. The ultrafast optical pump at 800nm, made up of 80fs pulses repeating at 1KHz (Coherent Astrella Laser system with TOPAS OPA), is reflected of a spatial light modulator (SLM) and the intensity profile from the image loaded on the SLM is projected onto the reconfigurable metasurface. The 800nm pump optical excites free carriers in the resonator and the quantum dots resulting in incoherent emission and refractive index change. We generate a 950nm (80fs pulse width and 1KHz repetition rate), by passing part of the 800nm pulse through an optical parametric amplifier (OPA) to generate an idler beam at 1900nm which is frequency doubled using a BBO-crystal, to only pump the QDs in the metasurface and estimate the temporal evolution of the PL. The light emission from the metasurface is imaged in the back-focal-plane using a single pixel (InGaAs detector) using lock-in amplifier setup. We measure the modulation signal at the sum frequency of the modulation of both 800nm and 950nm beam which modulated using a single chopper (5/7 relatively prime modulation) at 2 frequencies. The dual chop lock-in to the sum of the chopping frequency enables us to reduce the noise in the steering measurements[4,5,6]. We use a series of dichroic (830nm long pass at 45°), short-pass (1550nm short pass) and long-pass (1150nm, 1200nm long pass) filters to ensure that we are only collecting the PL signal from the metasurface. The PL directivity is measured by scanning the detector in the back-focal-plane for a given image projected onto the sample. Further details of the measurement setup and metasurface design can be found here[52,57].

Simulating incoherent emission from metasurfaces: Conventional Finite-Difference Time-Domain (FDTD) simulations that are used to model the behavior of metasurfaces rely on the assumption of coherent sources (with periodic boundary conditions), which limits interactions to coherent scattering processes. Point dipole sources, placed within resonators, enable us to predict the local density of photonic states and the Purcell enhancement for a single dipole. However, adding multiple dipoles with in the same simulation forces them to be coherent with respect to each other. Therefore, to model incoherent emission from a metasurface with a high density of spatially separated, mutually incoherent embedded quantum dot emitters, we need to perform independent FDTD simulations for each point dipole source. Within our metasurface, each nanopillar has 5 layers of high-(spatial) density InAs quantum dots (QDs) epitaxially grown with each layer having at least 30-40 independent quantum emitters – resulting in ~175-200 QD per

nanopillar. With our metasurface consisting of 750x750 (~500k), we estimate a total of ~ 98 million QDs which need to be independently simulated for each spatial index pattern projected onto the metasurface. This is a significant challenge, and we thus use closed-loop experimental feedback to discover governing equations, instead of relying on simulations.

2. Benchmarking the Machine Learning models

a. **Generative capability of the VAE**: The role of the generative model in our self-driving lab is to generate novel pump patterns beyond pump patterns currently explored in state-of-the-art experiments. In this work we use a VAE as our generative model, and to capture the generative capability of our VAE, we quantify the distribution of local slopes in the pump patterns generated by the VAE, see Figure S2. The local slope of a pump pattern y is defined as dy/dx, where x is the pixel index, i.e., the axis along which the intensity changes. Each pump pattern will thus have a distribution of local slopes, representing the variation in pump pattern intensity as a function of pixel location. Figure S2 shows that state-of-the-art sawtooth (grating order) pump patterns (green) have a narrow slope range, limited by assuming a fixed (sawtooth) functional form the pump patterns. The range of local slopes in the training set of the VAE (orange) is wider than the sawtooth patterns but is again limited by the assumption of a few fixed functional forms. The slopes of the patterns generated by the VAE (blue) are much broader than both the training set and the state-of-the-art sawtooth patterns, confirming that the VAE generates novel patterns beyond human intuition.

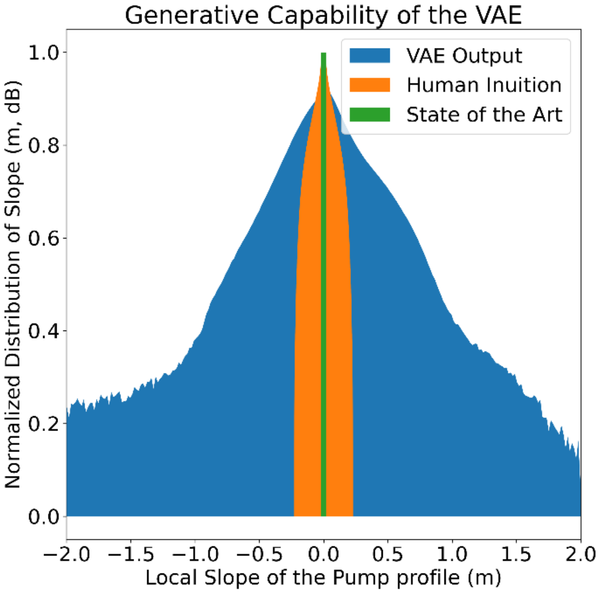

Figure S2: Generative Capability of the VAE. The normalized distribution (log scale) of the local slope (m) in the optical pump patterns imaged on to the metasurface. Green represents the saw-tooth patterns which were used in the state-of-the-art demonstration of incoherent emission steering. Orange represents human intuition-based VAE training set, while blue represents the local slope distribution from the output of the VAE.

Note that the VAE can generate pump patterns with large local slopes (over a few pixels). However, the sharpest feature realizable on the SLM is 30 pixels (3 microns), equivalent to 1% of the length of the pump pattern. This feature length is defined by the diffraction limit of the telescope imaging the SLM image to the metasurface at 800 nm. The sharpest patterns (high dy/dx) generated by the VAE which are beyond the capabilities of the experimental setup (due to diffraction limit) would be smoothed (blurred) out naturally by the propagation of the electromagnetic fields. In these scenarios, where the VAE pattern generates high spatial gradients, the active learning agent can only receive the experimental feedback with smoothed out pump patterns, thus predicting optimal pump patterns that account for these aberrations.

We also perform a preliminary t-SNE to reduce pump patterns to a lower dimension. We find that pump patterns that are known to affect steering can be reduced accurately to a low-dimensional space. While there is no clear separation of pump pattern features that correlate to high directivity, we see that families of pump patterns lie on low dimensional manifolds that effectively describe their structural features. Iterating over these complex low-dimensional features in pump patterns

directly relate to directivity, and our optimization method (active learning) navigates this search space to find patterns with high directivity.

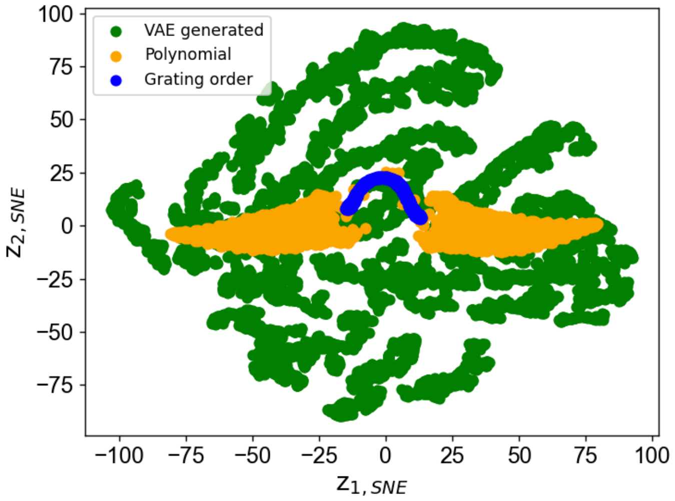

Figure S3: Latent space visualizations of families of pump patterns. Two-component latent space visualizations of grating order (saw-tooth) patterns (blue), patterns defined as polynomials (orange), and some patterns generated by sampling the VAE latent space (green), where the low-dimensional latent space descriptions are obtained by performing a t-SNE analysis on the pump patterns. We see that families of high-dimensional pump patterns lie on low-dimensional manifolds, supporting our leverage of the manifold hypothesis to generate candidate experiments.

b. **Efficiency of active learning:** The role of active learning in our self-driving lab is to discover pump patterns that have optimal performance, as measured by directivity. To benchmark active learning, we demonstrate a simple one-dimensional optimization, searching over the space sawtooth patterns with varying frequency (grating order) to find the sawtooth pattern with highest intensity at a specific angle. We choose this problem as prior work has a documented a solution, discovering that sawtooth pump patterns with grating order of +80 steer the most signal to a specific angle. This discovery was made using brute force iteration over 160 grating orders, and Figure S4 shows that active learning can re-discover this result using an order of magnitude (~20) experiments. In other words, active learning is an efficient way to guide experiments towards suitable pump patterns such that we discover optimal grating order patterns with minimal experiments, avoiding brute force parameter sweeps.

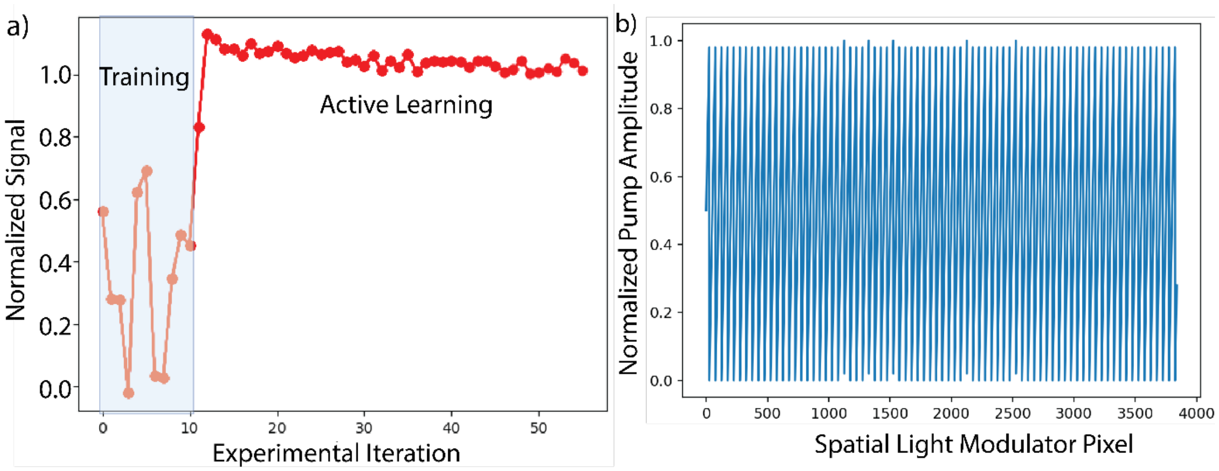

Figure S4. Efficiency of Active Learning for one dimensional problem. a) Normalized PL signal measured with the active learning optimizing over grating orders. Active learning is rediscovering a known result with 10% the number of experimental iterations required for a brute force search at an emission angle of -37◦. b)The plot shows the normalized pump amplitude at the end of the active learning process, corresponding to a grating order of 80.

3. Equation learner network fits and equations at other steering angles

Fig. S5 shows a parity plot visually documenting the quality of fits at various staging of the nn-EQL.

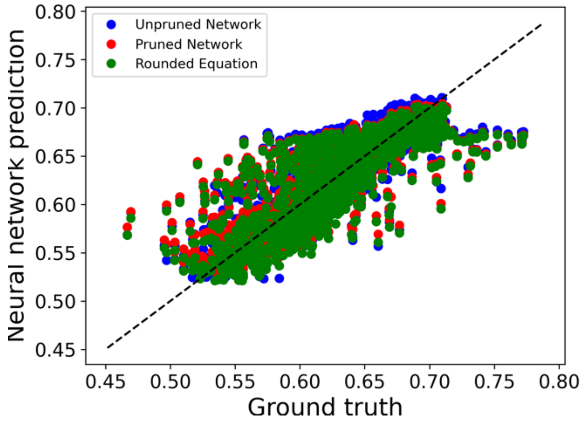

Figure S5. Parity plot from the nn-EQL. Parity plot showing nn-EQL's predictions at various stages of training, when compared to the ground truth directivity values. The average error after pruning (Stage 2) (red) and rounding of coefficients during equation read-out (Stage 3) (green) is higher than the error after initial training (Stage 1) (blue).

Also shown below are equations for other steering angles.

$$D_g(+14°) = -0.075a + 0.03b - 0.69(0.74\sin(x) - \sin(y))^2 + 0.055(-b + 0.3\sin(y))^2 - 0.33(0.02a + 0.13b + \sin(x) - 0.67\sin(y))^2 + 0.1(\sin(x) - \sin(y)) + 0.77 \quad (1)$$

where $x = 1.85\,a + 0.53\,b$ & $y = 2.09\,a + 0.15\,b$

$$D_g(-14°) = -0.02b - 0.26(0.55a - b + 0.152\sin(x))^2 + 0.054(a - 0.87b + 0.24\sin(x))^2 + 0.23(0.53a - b + 0.01\sin(x) - 0.65\sin(y))^2 + 1.11 \quad (2)$$

where $x = 2.16\,a + 1.2\,b$ & $y = 1.1\,a - 0.86\,b$

4. Steering over wide field of view with a refractive index profile consisting of a lens and grating

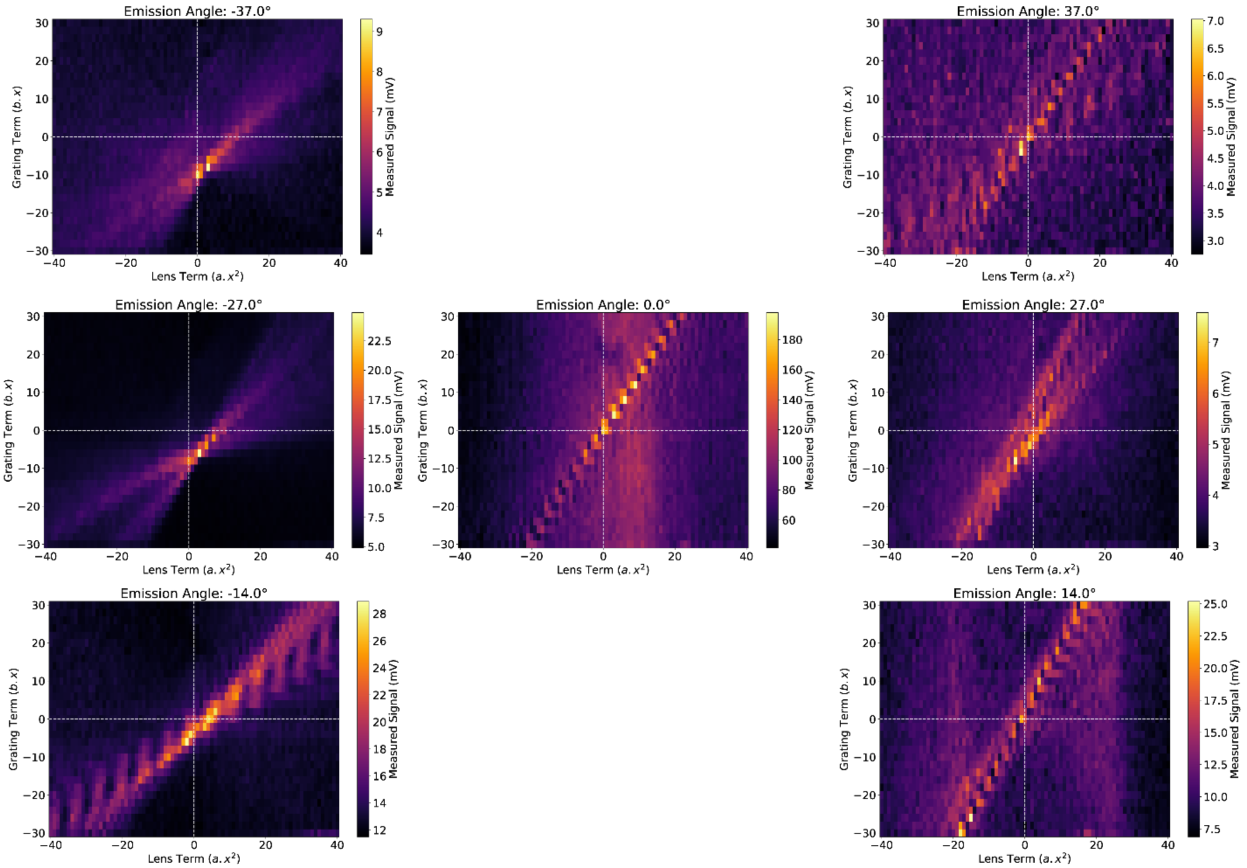

Figure S6: The far-field emission pattern from the metasurface under spatially structured optical pump formed as a combination of a lens ($a.x^2$) and a grating term (b.x) from -37° to + 37° with the negative angles on the left side and the positive angles on the right side.

References:

1. Bennett, B. & Soref, R. Electro refraction and electro absorption in InP, GaAs, GaSb, InAs, and InSb. IEEE J Quantum Electron 23, 2159–2166 (1987)

2. Adachi, S. Model dielectric constants of GaP, GaAs, GaSb, InP, InAs, and InSb. Phys Rev B 35, 7454–7463 (1987)

3. Prasankumar, R. P. et al. Ultrafast carrier dynamics in an InAs/InGaAs quantum dots-in-a-well heterostructure. Optics Express, Vol. 16, Issue 2, pp. 1165-1173 16, 1165–1173 (2008)

4. Quochi, F. et al. Coulomb and carrier-activation dynamics of resonantly excited InAs/GaAs quantum dots in two-color pump-probe experiments. Phys Rev B 67, 235323 (2003).

5. O'Driscoll, I. et al. Electron and hole dynamics of InAs/GaAs quantum dot semiconductor optical amplifiers. Appl Phys Lett 91, (2007).

6. Brener, I., Gershoni, D., Ritter, D., Panish, M. B. & Hamm, R. A. Decay times of excitons in lattice-matched InGaAs/InP single quantum wells. Appl Phys Lett 58, 965–967 (1991).
